# Sex-biased gene expression in the developing brain: implications for autism spectrum disorders

**DOI:** 10.1186/2040-2392-4-10

**Published:** 2013-05-07

**Authors:** Mark N Ziats, Owen M Rennert

**Affiliations:** 1 National Institute of Child Health and Human Development, National Institutes of Health, 49 Convent Drive, Building 49, Room 2C08, Bethesda, MD 20814, USA; 2 NIH – University of Cambridge Biomedical Scholars Program, Robinson College, Cambridge CB3 9AN, UK; 3 Baylor College of Medicine MSTP, One Baylor Plaza, Houston, TX 77030, USA

**Keywords:** Autistic disorder, Gene expression, Sex differences

## Abstract

Autism spectrum disorders affect significantly more males than females. Understanding sex differences in normal human brain development may provide insight into the mechanism(s) underlying this disparity; however, studies of sex differences in brain development at the genomic level are lacking. Here, we report a re-analysis of sex-specific gene expression from a recent large transcriptomic study of normal human brain development, to determine whether sex-biased genes relate to specific mechanistic processes. We discovered that male-biased genes are enriched for the processes of extracellular matrix formation/glycoproteins, immune response, chromatin, and cell cytoskeleton. We highlight that these pathways have been repeatedly implicated in autism and demonstrate that autism candidate genes are also enriched for these pathways. We propose that the overlap of these male-specific brain transcriptional modules with the same pathways in autism spectrum disorders may partially explain the increased incidence of autism in males.

## Correspondence

### Letter to the editor

Among the varied genetic, cellular, and clinical phenotypes described in autism spectrum disorders (ASD), the predominance of males versus females affected has stood apart as one of the most replicated findings. The most recent analysis indicates that at least four males are affected for every one female [1], and the male-to-female ratio is even higher among the least severely affected children with ASD [2]. Multiple hypothesis have been put forth to explain this phenomenon, including the notion that autistic children have brains that are more masculine [3-5]. Implicit in such theories is the idea that, by understanding the mechanism(s) contributing to the sex disparity of ASD, a better understanding of the underlying pathophysiology can be realized. While sex differences in ASD have mainly been studied at the behavioral level, molecular evidence that the autistic brain is somehow biased toward a male pattern of development has not been demonstrated.

Therefore, we were interested in assessing sex-biased patterns of normal human brain development at the genomic level, in an attempt to gain insight into processes that may underlie sex differences in ASD. We re-analyzed gene expression data from a recent whole-genome transcriptomics study of normal human brain development by Kang and colleagues [6]. This study performed genome-wide microarray analysis on unremarkable postmortem human brain tissue from 16 brain regions spanning preconception to adulthood. While Kang and colleagues’ study identified genes with male and female specific patterns of expression and highlighted individual genes of interest, we assessed them in aggregate to determine whether common biological pathways were over-represented. We used the gene ontology function DAVID 6.7 [7] to analyze all sex-biased genes together (that is, male versus female differentially expressed genes from all brain regions and developmental time points; see Additional file 1: Table S1). Additionally, we repeated this analysis using two other gene ontology databases: Ingenuity Pathways Analysis and GeneGo. Only functional ontologies with Benjamini–Hochberg multiple testing correction *P* <0.05 were considered significant.

The genes with female-biased expression patterns were not significantly enriched for any particular functional categories (Additional file 1: Table S2). However, genes with male-biased patterns of expression were enriched for the processes of: glycoproteins/extracellular matrix, immune response, nucleosome/chromatin, and cell cytoskeleton (for full list see Additional file 1: Table S3). Repeating the analysis using both Ingenuity Pathways Analysis and GeneGo yielded similar results (Additional file 1: Tables S4 to S7).

This finding is incredibly interesting in light of previous studies, as these pathways are consistently implicated in ASD. For instance, gene expression studies from postmortem autism brain tissue have demonstrated overexpression of immune response pathways in ASD [8,9]. Furthermore, both genetic and cellular studies have suggested that synaptogenesis is impaired in autism through cell adhesion, cell cytoskeleton, and cell–extracellular matrix binding pathways [10]. Moreover, recent large DNA sequencing studies repeatedly identified a chromatin-remodeling gene as one of only a few reaching genome-wide significance [11,12]. Interestingly, however, when we compared the list of genes with male-biased expression to those aberrantly expressed in ASD brain [8] or otherwise implicated in ASD [13], no genes overlapped. This suggests that perhaps individual genes themselves do not relate normal sexually dimorphic brain development to sex differences in ASD incidence, but rather suites of genes that funnel into common sex-specific brain development pathways may become perturbed in ASD.

To test this hypothesis, we also performed gene ontology enrichment analysis on autism candidate genes, to determine whether these male sex-biased pathways overlap with those implicated by ASD candidates. We assessed in aggregate putative autism genes curated in the AutKB database (both syndromic and nonsyndromic sets) [13]. We found that all of the male sex-biased pathways were also implicated by the ASD candidate genes (Additional file 1: Table S8). Taken together, these results demonstrate that pathways – but not the individual genes – implicated in autism overlap with normal male-specific transcriptional modules of the developing brain. We therefore propose that shared transcriptional modules, which influence both normal male brain development and the pathogenesis of ASD, may partially explain the increased incidence of autism in males.

Of unique interest is the finding of an immune system module in normal male-specific brain transcription. While peripheral immune responses have a significant gender dimorphism [14], the role of immune activation in ASD and its relation to sex differences has remained unclear. Based on a lack of enrichment for autism susceptibility genes in immune-related whole-genome co-expression networks that derived from genes differentially expressed in the autistic brain, Voineagu and colleagues concluded that immune findings in autistic brain are environmental rather than genetic [8]. However, our results suggest that a transcriptional program utilizing immune system components somehow may contribute to making a normal human brain more ‘male.’ To further explore our results in this context, we parsed the autism susceptibility genes into those that had been identified on the basis of microarray or proteomics studies (expression set) versus those that had been identified via studies assessing inherited or *de novo* DNA mutations (inherited set), such as genome-wide association, copy number variation, linkage, and other association analysis (see Additional file 1: Table S9). We discovered that immune-related modules were only significant in the expression set, but not the inherited set, of ASD candidate genes (see Additional file 1: Tables S10 and S11). It is then intriguing to speculate that while disruption of immune pathways in ASD may not be inherited, as Voineagu and colleagues propose and our results support, developing male brains may still be more susceptible to environmental insults than female brains because of their reliance on these immune-related pathways for normal male-specific brain development.

Overall, these results suggest the hypothesis that sex-specific transcriptional modules may make males more susceptible to neurodevelopmental disorders that result from aberrations – both inherited and environmental – in these pathways. As these four particular pathways are repeatedly implicated in ASD and overlap with pathways implicated by ASD candidate genes, we propose that normal sex differences in the functional genomics underlying human brain development may partly explain the significantly higher incidence of ASD in males. Notably, these four modules are more prominent in *normal* male versus female brain development, suggesting that any neurodevelopmental disorder resulting from aberrations in these pathways would be expected to have increased incidence in males. It will be important for future studies to further assess the role of male-specific gene expression programs as they relate to co-expression, dysregulation, and interaction with autism candidate genes in the developing brain, in addition to those of other neurodevelopmental and psychiatric disorders with sex-biased incidence.

## Abbreviations

ASD: autism spectrum disorders

## Competing interests

The authors declare that they have no competing interests.

## Authors’ contributions

MNZ and OMR conceived of and designed the analysis. MNZ performed the analysis. MNZ and OMR wrote the manuscript. Both authors read and approved the final manuscript.

## Supplementary Material

Additional file 1 **Supplementary data tables. Table S1** presenting genes differentially expressed by sex used for this analysis. **Table S2** presenting gene ontology results from DAVID analysis of female genes. **Table S3** presenting gene ontology results from DAVID analysis of male genes. **Table S4** presenting results from Ingenuity Pathways Analysis of male genes. **Table S5** presenting results from Ingenuity Pathways Analysis of female genes. **Table S6** presenting results from GeneGo analysis of male genes. **Table S7** presenting results from GeneGo analysis of female genes. **Table S8** presenting DAVID analysis of all AutKB Genes. **Table S9** presenting expression and inherited ASD candidate gene sets. **Table S10** presenting expression set DAVID enrichment results. **Table S11** presenting inherited set DAVID enrichment results. Click here for file
